# Counter-Intuitive Magneto-Water-Wetting Effect to CO_2_ Adsorption at Room Temperature Using MgO/Mg(OH)_2_ Nanocomposites

**DOI:** 10.3390/ma15030983

**Published:** 2022-01-27

**Authors:** Hasanthi L. Senevirathna, P. Vishakha T. Weerasinghe, Xu Li, Ming-Yan Tan, Sang-Sub Kim, Ping Wu

**Affiliations:** 1 Entropic Interface Group, Engineering Product Development, Singapore University of Technology and Design, 8 Somapah Road, Singapore 487372, Singapore; hasanthi_senevirathna@mymail.sutd.edu.sg (H.L.S.); puwakdandawe@mymail.sutd.edu.sg (P.V.T.W.); 2 Institute of Materials Research and Engineering, Agency for Science, Technology and Research (A*STAR), 2 Fusionopolis Way, Innovis, #08-03, Singapore 138634, Singapore; x-li@imre.a-star.edu.sg (X.L.); tanmy@imre.a-star.edu.sg (M.-Y.T.); 3 Department of Materials Science and Engineering, Inha University, Incheon 22212, Korea

**Keywords:** room temperature, CO_2_ adsorption, magneto-wetting, nesquehonite, aging

## Abstract

MgO/Mg(OH)_2_-based materials have been intensively explored for CO_2_ adsorption due to their high theoretical but low practical CO_2_ capture efficiency. Our previous study on the effect of H_2_O wetting on CO_2_ adsorption in MgO/Mg(OH)_2_ nanostructures found that the presence of H_2_O molecules significantly increases (decreases) CO_2_ adsorption on the MgO (Mg(OH)_2_) surface. Furthermore, the magneto-water-wetting technique is used to improve the CO_2_ capture efficiency of various nanofluids by increasing the mass transfer efficiency of nanobeads. However, the influence of magneto-wetting to the CO_2_ adsorption at nanobead surfaces remains unknown. The effect of magneto-water-wetting on CO_2_ adsorption on MgO/Mg(OH)_2_ nanocomposites was investigated experimentally in this study. Contrary to popular belief, magneto-water-wetting does not always increase CO_2_ adsorption; in fact, if Mg(OH)_2_ dominates in the nanocomposite, it can actually decrease CO_2_ adsorption. As a result of our structural research, we hypothesized that the creation of a thin H_2_O layer between nanograins prevents CO_2_ from flowing through, hence slowing down CO_2_ adsorption during the carbon-hydration aging process. Finally, the magneto-water-wetting technique can be used to control the carbon-hydration process and uncover both novel insights and discoveries of CO_2_ capture from air at room temperature to guide the design and development of ferrofluid devices for biomedical and energy applications.

## 1. Introduction

At present, renewable energy sources are garnering much attention due to their many advantages, including many environmental benefits [1]. However, in spite of the advantages, the majority of the energy requirements are fulfilled by utilizing fossil fuels. There are many adverse effects associated with anthropogenic activities, especially fossil fuel combustion, which contributes to the emission of carbon dioxide gas at an alarming rate. Compelling evidence supports the fact that the constant emission of CO_2_ gas into the atmosphere is the leading cause of global warming. Apart from that, accumulated gas in the atmosphere brings a series of adverse events such as air pollution, extreme weather conditions, etc. Hence, high levels of CO_2_ in the atmosphere are an alarming threat to all living beings in the world. Therefore, to mitigate these negative impacts, CO_2_ capture, and storage have gained the great interest of many research groups.

Consequently, the use of sorbent materials for carbon capture has received a great deal of attention. These materials include both solid and liquid-based materials. Solid adsorbents have its advantages over liquid adsorbents [2]. The use of solid adsorbents, such as metal organic frameworks (MOFs) [3,4,5,6], metal oxides [7,8,9,10], polymer-based sorbents [11,12,13], zeolites [14,15], etc., is now being heavily investigated. Although they perform well in CO_2_ adsorption, they require high temperature and/or pressure gradients to enable efficient sorption capacities. Particularly the use of magnesium hydroxide (Mg(OH)_2_) to produce magnesium oxide (MgO)-based adsorbents is gaining popularity due to a number of reasons, including its ability to capture CO_2_ at intermediate temperatures, low cost, availability, and non-toxicity [16,17]. Among the many advantages, Mg(OH)_2_ powder has been a widely used raw material in producing MgO for CO_2_ capture. Numerous experimental projects have been involved in investigating the production of Mg(OH)_2_ powder from the discharged brine from desalination plants [2,18,19,20], which has the potential to generate more economic and environmental benefits at the commercial scale production of MgO-based CO_2_ adsorbents.

Among many attempts to use pure MgO as a CO_2_ adsorbent, the capture capacities have been relatively low compared to other adsorbents mainly due to its fewer active sites to capture CO_2_ [21,22]. Apart from that, long-term CO_2_ fixation with MgO to produce thermodynamically stable magnesium carbonates (MgCO_3_) hinders further adsorption of CO_2_ on MgO. Gregg et al. [23] used pure MgO for CO_2_ adsorption and were able to obtain 0.4 mmol g^−1^, but since then, many studies have reported improved adsorption rates of pure MgO and MgO-based adsorbents at different conditions. MgO samples prepared by various methods affect the physical properties, which affects their performance in terms of CO_2_ capture. Subsequently, to improve the capture performance of MgO, various approaches have been reported [8,24,25,26,27,28,29].

The effect of an external magnetic field on sorbents for CO_2_ adsorption has been studied by many for different types of sorbent materials. The mass transfer enhancement using external magnetic fields has been studied [30,31,32]. In a study by Samadi et al. [30], a magnetic field was applied on Fe_3_O_4_/water ferrofluid to improve the CO_2_ absorption rate. The results showed that the absorption rate of CO_2_ in Fe_3_O_4_/water ferrofluid with 0.024 vol% under magnetic field is 21% higher than the case in which no magnetic field is applied. Recently, Khani et al. [32] reported on using a magnetic field as a new technique to increase the CO_2_ absorption in ferrofluids and reduce the pressure drop in a venturi scrubber. The study shows that under the magnetic field strength of 5120 G, increasing the nanofluid concentration from 0.01% to 0.05% led to an average increase of 20.5% in CO_2_ removal percentage and also to the use of 0.05 vol% Fe_3_O_4_/water nanofluid under a magnetic field with different strengths, significantly increasing the CO_2_ removal percentage in comparison with that of the distilled water. Darvanjooghi et al. [33] investigated the enhancement of the physical absorption of CO_2_ by Fe_3_O_4_ magnetic nanoparticles under the influence of AC and DC magnetic fields. According to their findings, the AC magnetic field increased CO_2_ solubility and the average molar flux of gas into the liquid phase. The diffusivity of CO_2_ in nanofluid and the renewal surface factor increased when the magnetic field intensity increased, resulting in a decrease in diffusion layer thickness.

The use of Fe_3_O_4_ particles in harvesting CO_2_ from aqueous solutions has been a topic of interest in recent times. Pahlevaninezhad et al. [34] recently evaluated the effect of Fe_3_O_4_ nanoparticles on the CO_2_–water mass transfer coefficient experimentally in the presence and absence of a magnetic field. This study shows that the mass transfer coefficient of CO_2_ in water and the effective mass transfer coefficient in nanofluid were increased by up to 10% and 29% in the presence of a parallel alternative magnetic field, respectively.

Other than Fe_3_O_4_ nanoparticles, the use of magnetic field effects for CO_2_ has been studied for other systems. Razmkhah et al. [35] reported on the effect of the external field on CO_2_ adsorption on the graphene oxide framework (GOF) studied by molecular dynamics simulation. They applied both an electric and magnetic field, parallel or perpendicular to the linker direction of the GOF and reported that there was no significant change in the GOF to CO_2_ adsorption by a magnetic field. Another study by Zhang et al. [36] presented the performance of ammonia-based CO_2_ adsorption under static magnetic field conditions, where the adsorption capacities were studied by a bubble reactor system. When comparing the absorption of CO_2_ under static magnetic field conditions and no magnetic field conditions, it was found that under the magnetic field, the absorption capacity of CO_2_ and the removal efficiency of CO_2_ were enhanced in ammonia-based sorbents. However, these studies presented the processes involving CO_2_ absorption assisted by a magnetic field instead of CO_2_ adsorption assisted by a magnetic field. Recently, the effect of magneto-water wetting on CO_2_ adsorption has been studied theoretically by Wu et al. [37]. This study replicated the phenomena of water-cluster fragmentation and hydrogen bond creation, which was reported by molecular dynamic simulation from 0 to 10 T, enabling a quantitative study of the magneto-wetting process. These studies suggest that there is a significant effect of magnetic fields on the CO_2_ adsorption efficiency with time.

In the current study, we report on the changes to the CO_2_ adsorption and the structural characteristics of the magnesium-based nanocomposite under a magnetic field. Despite the widespread use of ferrofluid devices in biomedical and industrial applications, where blood or aqueous CO_2_ levels are crucial to device efficiency, the related magneto-wetting effect for CO_2_ collection by ferro beans has yet to be investigated. In this work, we employed an experimental technique to examine this phenomena using solid adsorbents based on MgO/Mg(OH)_2_. We expected that by aging samples in a magnetic field, hydrates and carbonates would form as a CO_2_ barrier between nano-grains and slow the aging process at ambient temperature. We compare the sample data of aging under the magnetic field and without a magnetic field to hypothesize how the formation of a thin H_2_O layer deprives the CO_2_ adsorption. The findings demonstrated that the amount of CO_2_ adsorption of the samples changed under the influence of the magnetic field with time. The synthesized powder samples were characterized thoroughly using XRD, FE-SEM, BET, FTIR, and TGA analysis for their properties. The phase diagram and thermodynamic analysis were used to propose mechanisms for both thermal decomposition and CO_2_ adsorption under the influence of a magnetic field for a prolonged time.

## 2. Materials and Methods

### 2.1. Materials

For sample synthesis, analytical grade PVA (MW 89,000–98,000, 99+% hydrolyzed) and, as the precursor, Mg(OH)_2_ ≥99% (BioUltra) were purchased from Sigma-Aldrich (St. Louis, MO, USA). Analytical grade glacial Acetic acid (AA) 99.8% was purchased from Scharlau (Barcelona, Spain). All the chemicals were used without further purification. The water utilized in the experiments was deionized water (18 MΩ·cm).

### 2.2. Methods

The precursor solution for electrospinning was prepared by dissolving, 0.25 g of Mg(OH)_2_ in 5 mL of acetic acid under sonication in a water bath at 50 °C for 1 h until a clear solution was obtained. After that the aqueous PVA (5% *w*/*w*) solution, 0.750 mL was added to the clear solution and further sonicate in a water bath at 50 °C for 5 min to eliminate any precipitation. The electrospinning was then carried out in a similar manner as we reported in our earlier study [38]. The collected layer of nanofibers was kept drying at 60 °C for 48 h for drying. The oven-dried samples were then collected as solidified flakes and calcined in a muffle furnace (Nabertherm, Lilienthal, Germany) at 300 °C for 2 h at a rate of 2 °C min^−1^, naturally cooling to room temperature. The samples were then ground using a motor and pestle until we obtained a fine powder. The powder sample was then separated into two parts: one kept under a magnetic field (MF) with a strength of 175 Gauss for one month at room temperature and one in room temperature with no MF for one month to observe its effect to the aging.

## 3. Characterization

The X-ray diffraction (XRD) measurements of test samples were conducted using a D8 Advance ECO X-ray diffractometer (Bruker, Billerica, MA, USA) with Cu-Kα radiation of 1.54 Å to evaluate powder composition and phase. The scanning angle was adjusted between 2θ angles (from 10° to 70°) with the X-ray generator running at the applied voltage of 40 kV and current of 25 mA. Brunauer-Emmett-Teller (BET) analysis for surface area analysis was performed by using the ASAP 2020 system (Micrometrics, Norcross, GA, USA). The BET test was conducted at 120 °C using 0.1 g of powder samples. Fourier-transform infrared spectroscopy (FTIR) analysis was carried out using a Spectrum 2000 FTIR spectrophotometer (Perkin Elmer, Akron, OH, USA) in transmission mode ranging from 4000 to 400 cm^−1^ with a resolution of 1 cm^−1^. Surface structure and morphology were examined by using JSM-7600F field emission-scanning electron microscopy (FE-SEM) (JEOL, Tokyo, Japan). Thermogravimetric analysis (TGA) of the samples for CO_2_ capture and thermal decomposition of the aged samples was conducted using a TGA Q50 analyzer (TA Instruments, New Castle, DE, USA). The thermal decomposition analysis was carried out to obtain the time-dependent weight loss profile and phase transitions during the thermal decomposition of all three samples (before age, aged no MF, and aged MF).

## 4. Results Discussion

### 4.1. Evaluation of CO_2_ Capture Capacity

**Figure 1:**
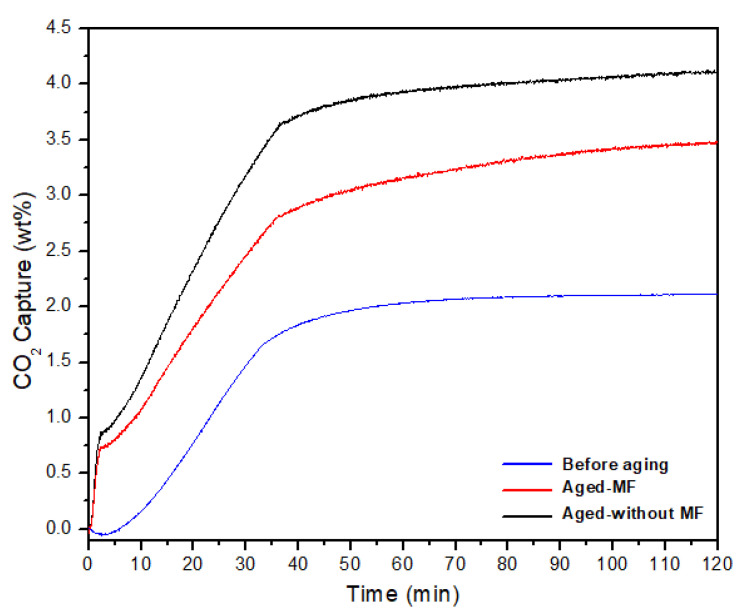
TGA data for electrospun MgO-based nanocomposites before aging, aged under MF, and aged without MF at room temperature.

For each sample, the CO_2_ adsorption capacity was measured using a Q50 TGA analyzer (Figure S1). TGA analysis for CO_2_ adsorption was done by loading 6–7 mg of samples on to a platinum (Pt) pan in the TGA unit. Samples were first pre-treated at 150 °C for 60 min under a flow of high purity N_2_ (40 mL min^−1^) with a ramp rate of 10 °C min^−1^. The temperature was then lowered to the 30 °C at a rate of 10 °C min^−1^ and the gas was switched from N_2_ to CO_2_ with a constant flow of pure CO_2_ (1 atm, 40 mL min^−1^). The sample was analyzed at 30 °C with a constant flow of high purity CO_2_ for 1.5 h as the longer time periods may not reveal any important information for practical applications [39]. Following Figure 1 shows the TGA data obtained for two samples kept under MF and one without MF.

As shown in Figure 1 regarding the sample before undergoing aging at room temperature, its CO_2_ adsorption at room temperature was around 2.12 wt%. The TGA data in Figure 1 also shows that both samples show an increase in CO_2_ adsorption from their original value, yet in comparison to the CO_2_ capture of the sample without MF (4.13 wt%), CO_2_ capture of the sample under the MF (3.48 wt%) shows less adsorption value at room temperature within 90 min after 1 month of natural aging at room temperature. This result indicates that there is an influence on CO_2_ adsorption under an MF for a long period of time. During the aging process, due to the atmospheric exposure, the sample tends to adsorb H_2_O and convert MgO in to Mg(OH)_2_ with time. However, according to Wu et al. [40], H_2_O molecules significantly facilitate CO_2_ capture on MgO but not on Mg(OH)_2_ and the formation of dehydration defects on Mg(OH)_2_ dramatically increases the CO_2_ adsorption energy from −0.045 eV to −1.647 eV. The optimized configuration of adsorbed CO_2_ on MgO surface and Mg(OH)_2_ surface as well as the optimized configuration in the presence of H_2_O molecules are shown in Figures S2 and S3.

### 4.2. Structural and Morphological Characterization

**Figure 2:**
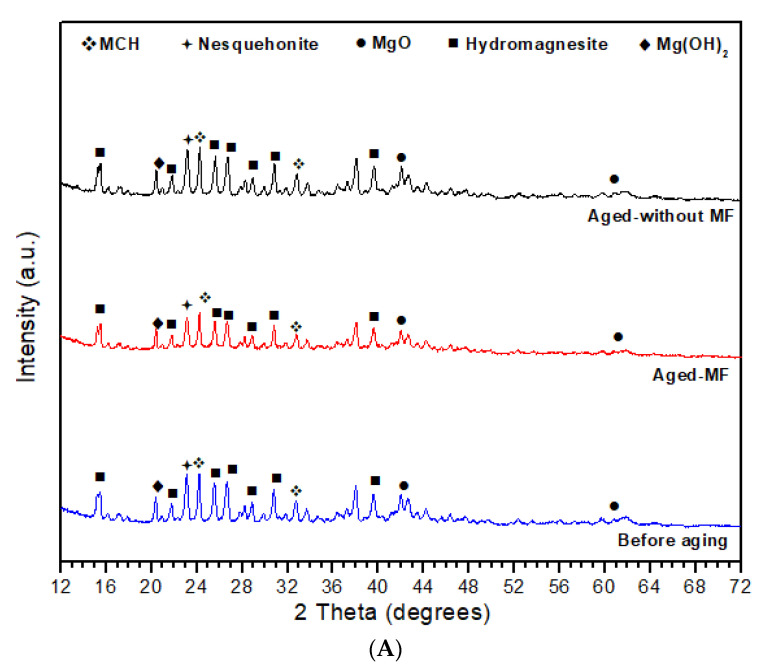

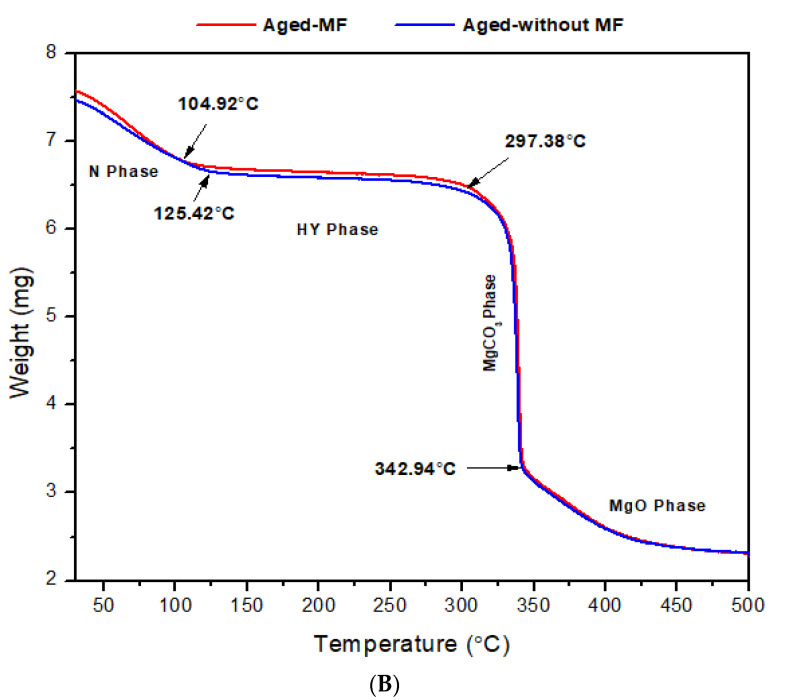
(**A**) XRD patterns for electrospun MgO-based nanocomposites, as prepared, after aging under MF and aging not under MF. (**B**) The thermal decomposition during the aging process under MF and no MF at room temperature.

To analyze the samples’ structural characteristics, XRD analysis and TGA analysis were carried out. Figure 2 presents the XRD and TGA decomposition analysis spectra for the samples before aging, after aging under MF, and under natural aging without MF for 1 month. The thermal decomposition of the samples was done using the TGA Q50 analyzer by setting the flow rate of the compressed dry air at 40 mL min^−1^ with a ramp rate of 10 °C min^−1^ from 30 to 500 °C. Samples (6–7 mg) were kept at 500 °C for 1 h.

Figure 2A shows the effect on MF to the structure with aging. The main difference observed is that the peak intensities of the sample aged under MF was reduced when compared to the no-MF sample. The original sample shows the presence of magnesium oxide (MgO), magnesium hydroxide (Mg(OH)_2_), Nesquehonite (N) (MgCO_3_.3H_2_O), Hydromagnesite (HY) (Mg_5_(CO_3_)_4_(OH)_2_·4H_2_O), and magnesium carbonate hydroxide hydroxides (MCH; Mg_2_CO_3_(OH)_2_·3H_2_O). The main 2θ peaks of the MgO, namely 42.58° and 61.80°, are consistent with (200) and (220), as well with the 2θ values of the residual Mg(OH)_2_ in 20.34°, consistent with the (001) lattice planes, which are in good agreement with MgO ICDD 01-071-6452 and Mg(OH)_2_ ICDD 01-082-2455. Other than that, 2θ peaks of Hydromagnesite, namely 15.31°, 21.71°, 25.51°, and 41.9°, are consistent with the (011), (210), (012), and (113) lattice planes of ICDD 00-025-051. 2θ peaks of MCH 24.14°, namely 32.72° and 39.65°, are consistence with the ($\bar{4}$01), (111), and (510) lattice planes of ICDD 00-006-0484. A peak related to Nesquehonite can be identified at 23.07° consistence with (002) lattice planes of ICDD 00-020-0669. Both aged with no MF and aged MF samples showed a slight peak shift in all the identified above peaks. However, the peak intensities of the sample aged under MF were less than that compared to the sample aged with no MF. A small amount of mismatch in peak positions was observed, which may have occurred due to an experimental error. Numerous other unidentified small peaks were observed in the samples, which may indicate the presence of residual polymer fragments from PVA used in electrospinning [38]. Some of the highly intensive peaks can be matched to several magnesium hydrates. However, the low intensity and large width peaks of samples can be explained by the fact that the H_2_O and CO_2_ molecules are easily chemisorbed onto the sample surfaces containing MgO when aging at room temperature [41]. Senevirathna et al. [38] explained the scenario of the sample aging under natural conditions for a long period of time where the sample is a nanocomposite, comprised of monoclinic magnesium malate tetrahydrate (C_8_H_10_MgO_10_⋅4H_2_O) or C8, nesquehonite, and residual MgO.

**Figure 3:**
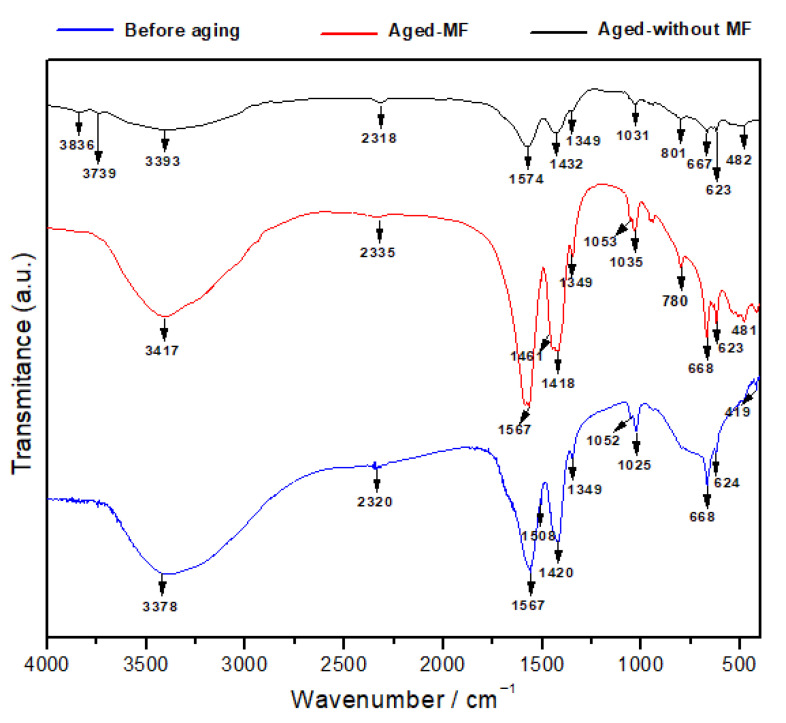
FTIR spectra for the samples as prepared: aging under MF and aging under no MF at room temperature.

Above Figure 2B shows the thermal decomposition of the aged samples under MF and without MF. According to the results, four different zones (Z1, Z2, Z3, and Z4) can be identified. The N-Phase (Z1), HY phase (Z2), MgCO_3_ phase (Z3), and MgO phase (Z4) were identified according to previously reported data. However, the Z3 can be observed to be rather minor compared to the other zones, indicating less MgCO3 in the samples. The thermal stability of the samples from 30 °C to 500 °C were obtained as Z2 > Z4 > Z1 > Z3. It was observed that for the sample aged under MF, it showed that its Z1 is in the range from 30 °C to 104.92 °C and Z2 from 104.92 °C to 297.38 °C, yet, for the sample aged without MF, it showed its Z1 from 30 °C to 125.42 °C and Z2 is in the range from 125.42 °C to 297.38 °C. For both samples, Z3 ranged from 297.38 °C to 342.94 °C and Z4 ranged from 342.94 °C to 500 °C. According to Dell and Weller [42], the initial weight loss may be due to the water loss in the N-phase and HY phases, where the HY phase has a comparatively large decomposition, as shown in Figure 3. Above 350 °C can be an appreciable amount of CO_2_ gas from carbonates and the residual weight reduction after 340 °C may correspond to the MgO content of N and HY [38,42]. The typical onset of decomposition of nesquehonite is from 70 °C to 100 °C and hydromagnesite is from 220 °C to 240 °C [43], which confirms the presence in the above Figure 2A,B, wherein XRD data confirms the presence.

The FTIR analysis for the samples was carried out to obtain additional information on their chemistry and structure, as shown in Figure 3. From FTIR analysis, the stretching vibration mode for the Mg–O–Mg compound was seen in the range of 481–668 cm^−1^, which is similar to what was reported in Balamurugan et al. [44] and Mohandes et al. [45] as strong and broad peaks in all three samples, while the as-prepared sample showed sharper and more intensive peaks than others. The sample under natural aging with no MF applied shows broader peaks for the stretching vibration mode for the Mg–O–Mg [44,45,46]. The stretching vibration of the O-H of water molecules and surface hydroxyl groups give rise to broad band in the region between 3378 cm^−1^, 3417 cm^−1^, and 3393 cm^−1^ in all three samples, yet the similar sample aged without MF shows a broad and less intensive peak related to the O-H stretching vibration (3393 cm^−1^) [47]. Two distinct bands are seen in the before-aging sample (1052 cm^−1^ and 1025 cm^−1^), aged sample under MF (1053 cm^−1^ and 1035 cm^−1^), and aged sample without MF (1031 cm^−1^ and 1024 cm^−1^), which are attributed to the bending vibration of absorbed water [44]. The peak intensity was reduced in the natural aged sample with no MF and increased the aged sample under MF, indicating more O-H formation. The difference is mainly due to the aerial adsorption of water molecules onto the MgO-based surfaces when they are exposed to the atmosphere where the sample under MF adsorbed more compared to the other two. The adsorption peak seen at the wavenumbers 1420 cm^−1^, 1418 cm^−1^, and 1432 cm^−1^ in all three samples can be assigned to the asymmetric stretching of the carbonate ion, which is of the CO_3_^2−^ species [47]. In addition, a weak band corresponding to the adsorption of gas-phase CO_2_ is visible at 2320 cm^−1^, 2335 cm^−1^, and 2318 cm^−1^ in all three samples [48,49]. However, the sample with natural aging with no MF applied shows weakened bonds. When compared to the fresh sample, due to the CO_3_^2−^ chemisorption with natural aging, a shift is observed towards the higher wavenumbers [41]. Interestingly, the peak intensity of all the peaks identified are reduced in the natural-aged sample with no MF. The peak at 1567 cm^−1^ and 1574 cm^−1^ is attributable to the influence of the C=O vibration of CO_3_^2−^ [49], where the aged-under-MF sample shows an intensive peak compared to the no-MF sample, indicating that under MF, the formation of CO_3_^2−^ increased in the sample.

### 4.3. Morphology Analysis

In order to have an idea of the effect on the surface area of the samples under MF, BET analysis was carried out. The surface area and pore volume parameters of samples are shown in Table 1.

The synthesized samples had a higher pore volume but less surface area before aging at room temperature. In particular, the BET surface area reached as high as 79.52 m^2^/g when it aged without MF for 1 month at ambient conditions, yet it recorded the lowest pore size as 3.48 nm. When compared with the sample aged with no MF, the sample aged under MF shows a lesser surface area of 25.62 m^2^/g, yet it shows a higher pore size of 216.93 m^2^/g. This result indicates that there is an effect of the MF to the sample’s microstructure. This result can be validated using the TGA results obtained where the highest CO_2_ adsorption at room temperature was recorded for the sample aged without MF, which records the high BET surface area of the three samples.

**Figure 4:**
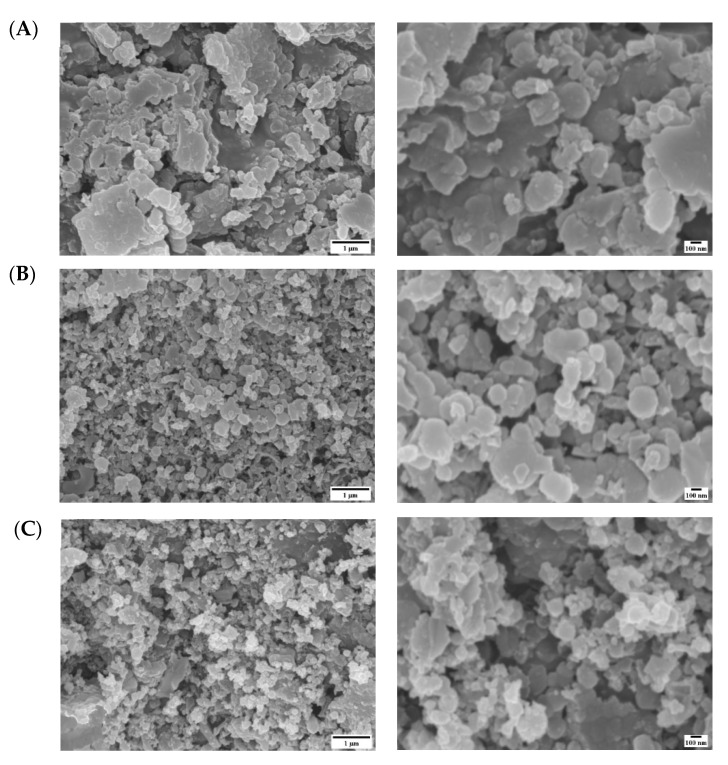
SEM images for (**A**) sample as prepared. (**B**) Sample aged under no MF. (**C**) Sample aged under MF for 1 month at room temperature. Right side indicates low magnification and left side high magnification.

The surface analysis of the samples was carried out using SEM analysis. Figure 4 presents the SEM images obtained for three samples.

All the samples show sheet like structures, yet the aged samples show more uniform and smooth structures at the nanoscale features, varying from 100–200 nm.

## 5. Discussion

**Figure 5:**
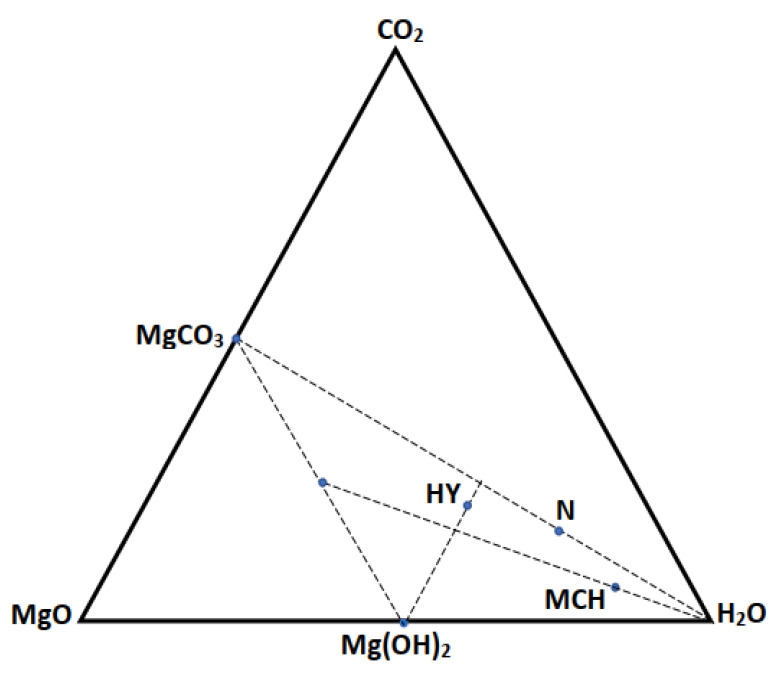
Phase relationship for the studied system.

Based on the data collected following Figure 5, we summarize the mechanism to explain the relationship of our sample with aging under MF and without MF. H_2_O molecules govern the volume ratio between CO_2_-phobic (Mg(OH)_2_) and CO_2_-phylic (MgO) grains, and the total volume of Mg(OH)_2_ rises as the quantity of H_2_O molecules grows according to prior density-functional theory (DFT) calculations [40]. Additional H_2_O molecules may precipitate between the MgO and Mg(OH)_2_ grains due to magneto-wetting in this work, forming H_2_O thin films that inhibit CO_2_ molecules from diffusing between the MgO and Mg(OH)_2_ grain boundaries, which is consistent with the aforementioned notion. As a result, the popular belief that the magneto-water-wetting effect increases the CO_2_ collection efficiency of diverse nanofluids is incorrect.

The control mechanism of magnetic radiation is the formation of an H_2_O film on the surface of pores due to the magneto-wetting [37]. This H_2_O film hinders the diffusion of vapor-H_2_O into the lattice and slows the hydration of the powder during the aging process. The as-prepared powder sample is located along the MgO to H_2_O line in Figure 5. However, with prolonged aging, it goes towards MgCO_3_, which quickly formed at 342.94 °C (Figure 3). The Z2 zone has a long temperature range to form HY, which may be due to the formation of MCH and N simultaneously (Figure 2B). After 125.42 °C, MCH and N form. For continuous aging, the sample may follow the line linking MCH, N, and CO_2_ in the diagram in Figure 5. It has been established that the MF could affect the physicochemical properties of water [50,51,52,53] as, under the MF, water molecules become magnetized. Han et al. [54] reported on the effect of MF on the optical property states that the spiral motion of ions is imposed to the water molecules due to the MF. Holysz et al. [51] concluded that MF may increase the electrolyte conductivity due to the decrease in ion radius. Fujimura and Iino [55] reported on an increase in surface tension with an increase of MF due to the stabilization of hydrogen bonds. The surface tension increases when the bulk Helmholtz free energy increases due to the stabilized hydrogen bonds. As the sample ages at room-temperature conditions, atmospheric H_2_O and CO_2_ molecules tend to adsorb the samples, resulting in a change in the samples over time [38]. Cai et al. [52] reported that the water intramolecular energy decreased, and activation energy increased over the exposure time of MF to the sample, and the results suggested that more hydrogen bonds were formed, and the mean size of water clusters got larger by magnetic field treatments to the samples. Therefore, due to these arrangements, more H_2_O molecules tend to adsorb into sample under the MF, resulting in H_2_O molecules blocking the pores and leading to the relative decrease in CO_2_ adsorption in comparison with the aged samples without MF. This can also promote the formation of carbonates in the samples, hence hindering the adsorption (Figure 2A).

## 6. Conclusions

The effect of magneto-wetting on CO_2_ adsorption was experimentally investigated using nano-MgO/Mg(OH)_2_-based materials. Ambient H_2_O contributes to the Mg(OH)_2_ nucleation and growth process in a MgO/Mg(OH)_2_ composite during normal aging. However, when an MF is present, the Mg(OH)_2_ growth process is accelerated and additional H_2_O molecules may be trapped between the grains of MgO and Mg(OH)_2_ to further impede CO_2_ diffusion. We demonstrated that, contrary to popular belief, magneto-water-wetting does not always increase CO_2_ adsorption; in fact, when Mg(OH)_2_ predominates in the nanocomposite, CO_2_ adsorption might actually decrease. As a result, the impact of MF on CO_2_ adsorption is critical. These discoveries might lead to applications such as extending the life of CO_2_ adsorbents and controlling the hydration process at room temperature. The findings might be used to design and develop new nanofluid devices for medical and industrial applications in which blood CO_2_ levels are critical for device efficiency.

## Figures and Tables

**Table 1:** Effect of MF for surface area and pore volume parameters of samples.

Sample	Surface Area (m^2^/g)	Total Pore Volume (cm^3^/g)	Avg Pore Size (nm)
As prepared	12.45	0.359	115.60
Aged no MF	79.52	0.069	3.48
Aged under MF	25.62	1.389	216.93

## Data Availability

The data presented in this study are available upon request from the corresponding author.

## Supplementary Materials

The following supporting information can be downloaded at: https://www.mdpi.com/article/10.3390/ma15030983/s1, Figure S1: A schematic diagram of the TGA analysis process for the CO_2_ capture capacity of the samples and figure of TGA Q50 analyser [1]; Figure S2: Optimized configuration of adsorbed CO_2_ on MgO surface (a) and with H2O (b). Atoms are shown as colored balls: hydrogen (white), oxygen (red), magnesium (green), and carbon (grey); Figure S3: Optimized configuration of adsorbed CO_2_ on Mg(OH)2 surface (a) and with H2O (b). Atoms are shown as colored balls: hydrogen (white), oxygen (red), magnesium (green), and carbon (grey).
