# Supplementary material for: Counter-Intuitive Magneto-Water-Wetting Effect to CO2 Adsorption at Room Temperature Using MgO/Mg(OH)2 Nanocomposites
Source: Materials (Basel). 2022 Jan 27;15(3):983. doi: 10.3390/ma15030983 (PMC8838735; doi:10.3390/ma15030983)
Supplement: Supplementary file 1 [file materials-15-00983-s001.zip › materials-1539980-supplementary.pdf]

materials

MDPI

Article

Counter-Intuitive Magneto-Water-Wetting Effect to CO_2 Adsorption at Room Temperature Using $MgO/Mg(OH)_2$ Nanocomposites

Hasanthi L. Senevirathna [1], P. Vishakha T. Weerasinghe [1], Li Xu [2], Ming-Yan Tan [2], Sang-Sub Kim [3,*] and Ping Wu [1,*]

[1] Entropic Interface Group, Engineering Product Development, Singapore University of Technology and Design, 8 Somapah Road, Singapore 487372, Singapore; hasanthi_senevirathna@mymail.sutd.edu.sg (H.L.S.); puwakdandawe@mymail.sutd.edu.sg (P.V.T.W.)
2 Institute of Materials Research and Engineering, Agency for Science, Technology and Research (A*STAR), 2 Fusionopolis Way, Innovis, #08-03, Singapore 138634, Singapore; x-li@imre.a-star.edu.sg (L.X.); tanmy@imre.a-star.edu.sg (M.-Y.T.)
[3] Department of Materials Science and Engineering, Inha University, Incheon 22212, Korea
* Correspondence: sangsub@inha.ac.kr (S.-S.K.); wuping@sutd.edu.sg (P.W.)

Abstract: $MgO/Mg(OH)_2$-based materials have been intensively explored for CO_2 adsorption due to their high theoretical but low practical CO_2 capture efficiency. Our previous study on the effect of H_2O wetting on CO_2 adsorption in $MgO/Mg(OH)_2$ nanostructures found that the presence of H_2O molecules significantly increases (decreases) CO_2 adsorption on the MgO ($Mg(OH)_2$) surface. Furthermore, the magneto-water-wetting technique is used to improve the CO_2 capture efficiency of various nanofluids by increasing the mass transfer efficiency of nanobeads. However, the influence of magneto-wetting to the CO_2 adsorption at nanobead surfaces remains unknown. The effect of magneto-water-wetting on CO_2 adsorption on $MgO/Mg(OH)_2$ nanocomposites was investigated experimentally in this study. Contrary to popular belief, magneto-water-wetting does not always increase CO_2 adsorption; in fact, if $Mg(OH)_2$ dominates in the nanocomposite, it can actually decrease CO_2 adsorption. As a result of our structural research, we hypothesized that the creation of a thin H_2O layer between nanograins prevents CO_2 from flowing through, hence slowing down CO_2 adsorption during the carbon-hydration aging process. Finally, the magneto-water-wetting technique can be used to control the carbon-hydration process and uncover both novel insights and discoveries of CO_2 capture from air at room temperature to guide the design and development of ferrofluid devices for biomedical and energy applications.

Keywords: room temperature; CO_2 adsorption; magneto-wetting; nesquehonite; aging

Citation: Senevirathna, H.L.; Weerasinghe, P.V.T.; Li, X.; Tan, M.-Y.; Kim, S.-S.; Wu, P. Counter Intuitive Magneto-Water-Wetting Effect to CO_2 Adsorption at Room Temperature Using $MgO/Mg(OH)_2$ Nano Composites. *Materials* **2022**, *15*, 983. https://doi.org/10.3390/ma15030983

Academic Editor: Paula Teixeira

Received: 20 December 2021
Accepted: 19 January 2022
Published: 27 January 2022

Publisher's Note: MDPI stays neutral with regard to jurisdictional claims in published maps and institutional affiliations.

Copyright: © 2022 by the authors. Licensee MDPI, Basel, Switzerland. This article is an open access article distributed under the terms and conditions of the Creative Commons Attribution (CC BY) license (https://creativecommons.org/licenses/by/4.0/).

Supplement Materials

TGA analysis was done by loading 6–7 mg to a platinum pan in the TGA unit, for CO_2 adsorption performance measurement. To avoid errors caused by pre-adsorbed species as atmospheric CO_2, water, and other impurities, samples were first pre-treated at 150 °C for 60 min under a flow of high purity N_2 (40 mL min^{-1}) with a ramp rate of 10 °C min^{-1}. The temperature was then lowered to the desired adsorption temperature at a rate of 10 °C min^{-1}, the gas was switched from N_2 to CO_2 with a constant flow of high purity CO_2 (1 atm, 40 mL min^{-1}), and the CO_2 adsorption uptake was measured for 90 min.

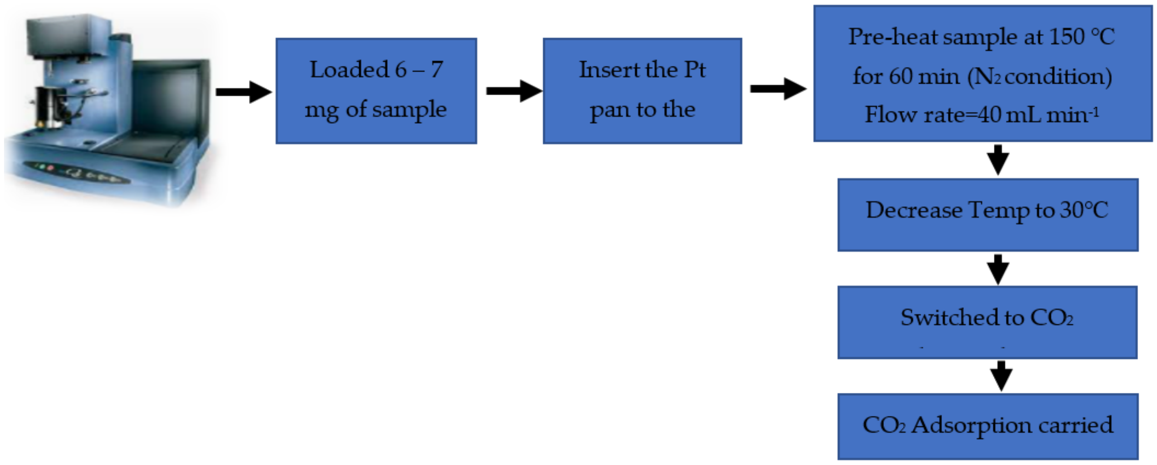

Figure S1. A schematic diagram of the TGA analysis process for the CO_2 capture capacity of the samples and figure of TGA Q50 analyser [1].

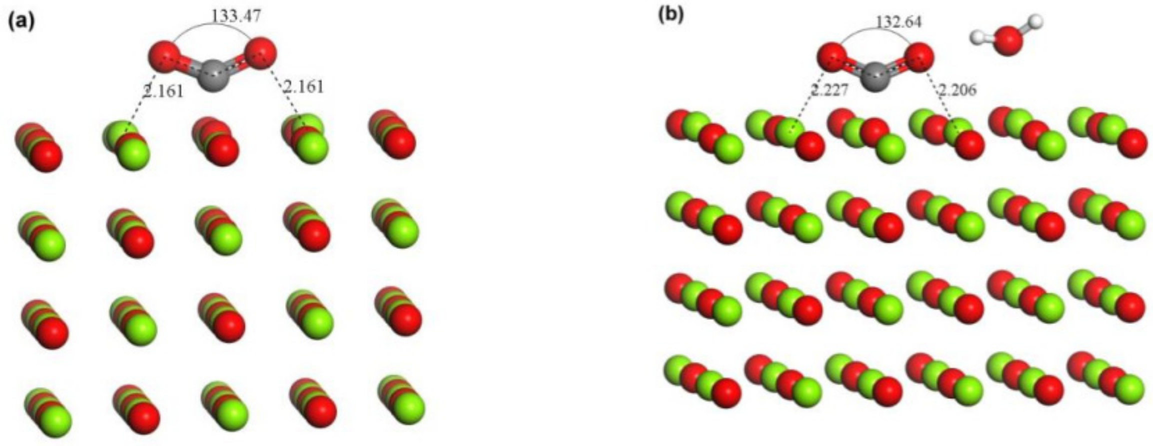

Figure S2. Optimized configuration of adsorbed CO_2 on MgO surface (**a**) and with H_2O (**b**). Atoms are shown as colored balls: hydrogen (white), oxygen (red), magnesium (green), and carbon (grey).

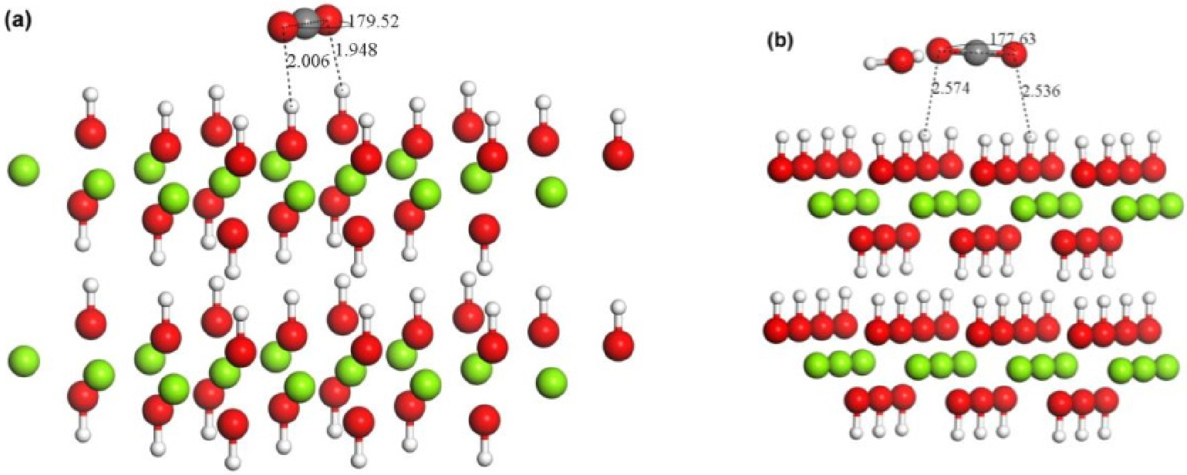

Figure S3. Optimized configuration of adsorbed CO_2 on $Mg(OH)_2$ surface (**a**) and with H_2O (**b**). Atoms are shown as colored balls: hydrogen (white), oxygen (red), magnesium (green), and carbon (grey).

Reference

1. TA Instruments. TA instruments thermogravimetric analyzers. Available online: http://www.tainstruments.com/pdf/literature/OLD_tgabrochure.pdf (accessed on 5 January 2022).
